# Case report: Autoimmune encephalomyelitis following cytomegalovirus infection after allogeneic hematopoietic stem cell transplantation

**DOI:** 10.3389/fmed.2024.1373062

**Published:** 2024-05-30

**Authors:** Min Yang, Yu Cai, Liping Wan, Linhua Ji, Xian M. Song

**Affiliations:** ^1^Department of Hematology, Shanghai General Hospital, Shanghai, China; ^2^Shanghai Jiao Tong University School of Medicine, Shanghai, China; ^3^Huadu District People’s Hospital of Guangzhou, Guangzhou, China

**Keywords:** autoimmune encephalomyelitis, allogeneic hematopoietic stem cell transplantation, human cytomegalovirus, high-dose steroids, intravenous immunoglobulins

## Abstract

**Introduction:**

Cytomegalovirus (CMV) can cause various end-organ diseases in immunocompromised hosts, including allogeneic hematopoietic cell transplant (allo-HSCT) recipients. Interestingly, CMV viremia has been associated with various complications and poor prognosis in allo-HSCT recipients. Complications involving the central nervous system (CNS) occur in 9–14% of patients following allo-HSCT. However, autoimmune encephalitis (AE) secondary to CMV infection after allo-HSCT has rarely been reported. Here we report a case of possible AE following CMV viremia after allo-HSCT, which was successfully treated with high-dose pulsed methylprednisolone and intravenous immunoglobulins (IVIg).

**Case description:**

A 53-year-old female underwent allo-HSCT for T-lymphoblastic lymphoma/leukemia. The patient developed CMV viremia on day 36 after transplantation, and serum CMV-DNA remained positive after initiating ganciclovir antiviral therapy, turning negative one month later. Four months later, she started experiencing memory impairment, weakness in the left limbs, cognitive dysfunction, and hallucinations. A magnetic resonance imaging brain scan showed scattered ischemic lesions under the bilateral frontal cortex. Viral detection in cerebral spinal fluid (CSF) by next-generation gene sequencing technology showed no obvious abnormality. Antibodies specific to AE and paraneoplastic diseases in serum and CSF were absent. The oligoclonal bands in the CSF were detected using isoelectric focusing and immunofixation, and the results were negative. However, after extensive investigation regarding infections, autoimmune disorders, and recurrence of the malignancy, possible AE could not be excluded. The patient was treated with high-dose steroids combined with IVIg therapy; the patient’s symptoms were significantly improved.

**Conclusion:**

The mechanisms of AE after allo-HSCT and the relationship with CMV infection should be further studied. Therefore, reporting this and similar cases will improve our awareness and understanding of the underlying disease mechanisms.

## 1 Introduction

Cytomegalovirus (CMV) can cause various end-organ diseases in immunocompromised hosts, particularly allogeneic hematopoietic stem cell transplant (allo-HSCT) recipients (1). Accordingly, CMV viremia has been associated with several complications and poor prognoses in allo-HSCT recipients (2). Complications involving the central nervous system (CNS) (3), including encephalitis, stroke-like episodes, demyelination, and non-specific neurological symptoms, occur in 9–14% of patients following allo-HSCT. However, autoimmune encephalitis (AE) secondary to CMV infection after allo-HSCT is rare. Consequently, the specific mechanisms by which CMV infection after allo-HSCT induces autoimmune encephalitis are unclear. Therefore, reporting this and similar cases will help clinicians in their diagnosis and treatment of AE and improve our awareness and understanding of the underlying disease mechanisms.

## 2 Case description

A 53-year-old Chinese woman was diagnosed with T-lymphoblastic lymphoma/leukemia after a pathological biopsy of the lymph nodes on the right side of her neck. Bone marrow remission was achieved after induction therapy. Whole-body positron emission tomography (PET)–computed tomography (CT) eventually indicated complete remission after six cycles of chemotherapy (Supplementary Table S1). The patient subsequently underwent allo-HSCT to treat the high-risk T-lymphoblastic lymphoma/leukemia. Lumbar puncture revealed no evidence of leukemic invasion of the CNS before transplantation. The patient received human leukocyte antigen proteins for allo-HSCT from a half-matched donor (Supplementary Table S2). Her conditioning regimen consisted of the following: cyclophosphamide, 100 mg/kg on days −5 to −4, etoposide, 20 mg/kg on day −5 to −4, total body irradiation, 3 GY on days −3 to −1, rabbit anti-human thymocyte immunoglobulin, 5.2 mg/kg on days −2 to −1; cyclosporin A, 70 mg/day from day +4; and cyclophosphamide, 50 mg/kg on day +4. The patient received mycophenolate sodium enteric-coated tablets (720 mg/day on days +4 to +34) for prophylaxis against graft-versus-host disease (GVHD), and oral valacyclovir (1.2 g/day) and posaconazole (0.3 g/day) for prevention of viral and fungal infections, respectively. Neutrophils were implanted on day +10.

## 3 Diagnostic assessment

On the 33rd day after transplantation, CMV-DNA was detected in the patient’s serum (3,303 copies/mL). Ganciclovir was administered (5 mg/kg/day); however, serum tests for CMV-DNA remained positive until gradually turning negative after continuing antiviral treatment for 1 month. On day 119 after allo-HSCT, the patient developed short-term memory dysfunction and emotional lability. The patient exhibited weakness in the upper left and lower left limbs on day 163 after transplantation but did not report any paresthesia. Consequently, the symptoms persisted for 1 day before the patient was admitted to the emergency department of our hospital.

**Figure 1:**
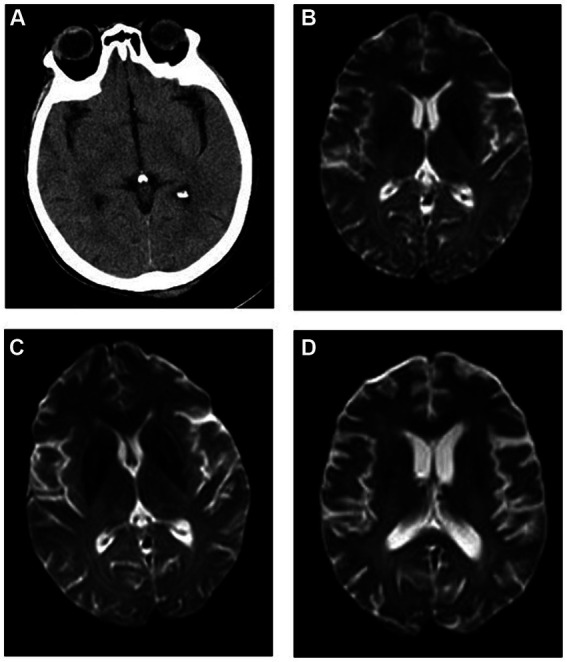
Imaging findings. **(A)** Brain computed tomography (CT) of the patient on onset of autoimmune encephalitis (AE) revealing relatively normal findings, and cranial magnetic resonance imaging (MRI) **(B)** showing scattered ischemic lesions under the bifrontal cortex. Cranial MRI at **(C)** 1 and **(D)** 6 months (1 year after allogeneic hematopoietic cell transplant) after AE onset showing no significant changes when compared with previous imaging findings.

The brain CT scans of the patient were normal. According to the International Classification of Diseases, 11th Revision (4), if imaging does not provide evidence of the responsible lesion, the duration of symptoms and signs exceeding 24 h is still used as the time limit for diagnosing ischemic stroke. Given the narrow treatment time window for acute ischemic stroke, timely evaluation and treatment are crucial. The patient was empirically treated with aspirin (0.1 g/day), clopidogrel (0.075 g/day) for antiplatelet aggregation, butylphthalide (0.05 g/day) to improve collateral circulation, edaravone (0.02 g/day) to scavenge free radicals, and atorvastatin (0.01 g/day) to regulate blood lipid levels. Cranial magnetic resonance imaging (MRI) conducted 3 days later showed scattered ischemic lesions under the bifrontal cortex (Figure 1). After treatment, the patient experienced minor symptom relief. Despite good initial recovery, the patient was admitted to the emergency department of our hospital on day +185 with hallucinations and significant memory issues, which manifested as ecmnesia with remote amnesia. Physical examination revealed that the patient was conscious but unresponsive, had ambiguous speech, and was unable to add and subtract numbers ≤10. Left upper and left lower limb muscle strengths were of grades 3 and 2, respectively. No other physical signs of fits, paralysis, or focal neurologic symptoms were observed.

Although protein levels were mildly elevated (0.83 g/L), no viruses (CMV, Epstein–Barr virus, herpes simplex virus, or varicella zoster) were detected via metagenomic next-generation sequencing of the cerebrospinal fluid (CSF). The oligoclonal bands in the CSF were detected using isoelectric focusing and immunofixation, and the results were negative. Both AE and paraneoplastic antibody testing of the peripheral blood and CSF via indirect immunofluorescent testing and western blotting failed to reveal any apparent abnormalities. The dosage of cyclosporin A was reduced. However, no improvement in the patient’s general status was observed. Electroencephalogram findings were normal without epileptiform discharge.

**Figure 2:**
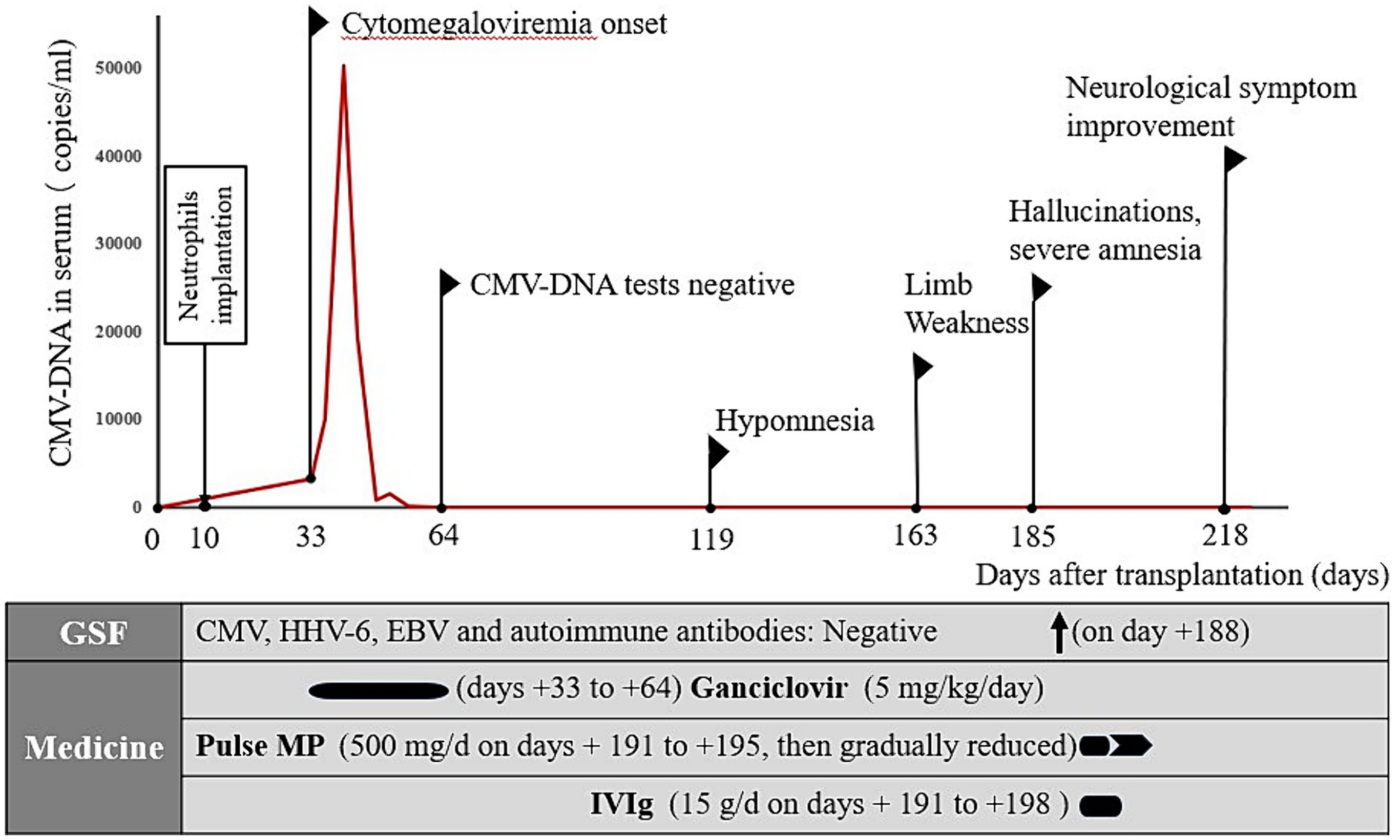
Summary of the disease course. CMV, cytomegalovirus; HHV-6, human herpesvirus-6; EBV, Epstein–Barr virus; MP, methylprednisolone; IVIg, intravenous immunoglobulin.

After comprehensive evaluation of the patient’s condition and auxiliary examinations, the diagnosis of possible AE was determined. The patient received IVIg (15 g/day on days 1–8) and methylprednisolone (500 mg/day on days 1–5, 240 mg/day on days 6–10, and 120 mg/day on days 11–15). Intravenous methylprednisolone was discontinued on day 16 and methylprednisolone tablets were administered orally at 60 mg/day. Cranial MRI performed after treatment showed no significant changes compared with prior imaging findings (Figure 1). However, the patient’s consciousness, cognitive function, and muscle strength significantly improved (Figure 2).

One year after allo-HSCT, cranial MRI (Figure 1) and whole-body PET–CT revealed no obvious abnormalities. The patient’s cognitive function and muscle strength in the left upper and left lower limbs had recovered; however, mild deficits in attention and memory persisted 1 year after allo-HSCT.

## 4 Discussion

This case describes the successful treatment of possible AE due to CMV infection following allo-HSCT. Our patient presented with memory impairment, cognitive dysfunction, hallucinations, and weakness in the left limbs after transplantation; however, no obvious abnormalities were observed on brain CT or MRI. Further, no viruses or antibodies specific to AE or paraneoplastic diseases were noted in the peripheral blood and CSF. Testing for oligoclonal bands in the CSF yielded negative results. The patient was infected with CMV prior to symptom onset; however, no CMV was detected in the CSF by metagenomic next-generation sequencing. We suspect that the AE after allo-HSCT may be associated with CMV infection (5). The correlation between AE after allo-HSCT and CMV infection has not been fully clarified. Thus, pathogenicity and disease specificity require further investigation.

CNS involvement following CMV infections is rare, particularly in the form of AE. Therefore, distinguishing between AE and its differential diagnoses, especially other infectious complications, cerebral lymphoma, or cerebrovascular accident, is crucial. Additionally, some rare CNS complications after HSCT should be excluded. Acute GVHD is a major life-threatening complication after allo-HSCT. GVHD can also affect the CNS as a major immune-mediated side effect (6, 7). Clinical manifestations of CNS-GVHD include seizures, vision loss, aphasia, and cognitive impairment. The symptoms are similar to multiple sclerosis or Guillain–Barre syndrome (6, 8). Transplantation-associated thrombotic microangiopathy (TA-TMA) has been shown to be another rare potential neurological complication of allogeneic stem cell transplantation (9). Luo et al. conducted a retrospective analysis of 206 children after allo-HSCT and found that five children had CNS symptoms, including convulsion and drowsiness (10). The procedure for differential diagnoses was performed on our patient after repeated imaging and extensive CSF testing, considering her medical history and physical signs. Therefore, we ruled out other diagnoses, such as multiple sclerosis (11), acute myelitis, cerebral hemorrhage, cerebral tumor, and GVHD and TA-TMA-related CNS complications.

The annual number of allo-HSCTs performed as potential curative treatments for malignant hematological diseases has steadily increased over recent years (12). However, allo-HSCT remains fraught with complications including GVHD and opportunistic infections. CMV infection is a common, potentially life-threatening complication that typically occurs after allo-HSCT (13). CMV is a member of the beta-herpesvirus family. This family of viruses has established lifelong latent infections in >70% of the human population (14). Under appropriate conditions, recurrence with life-threatening replication rates occurs, particularly in patients undergoing allo-HSCT with abrogated T-cell immunity (13, 15). CMV infection in HSCT recipients occurs most commonly in the early post-HSCT period (< 100 days), corresponding to the period of the greatest degree of immunosuppression. In this case, CMV-DNA was detected in the serum on the 33rd day after transplantation and gradually disappeared after 1 month of antiviral treatment.

CMV can infect many organs including the kidneys, pancreas, intestines, liver, heart, lungs, tissue allografts, and blood cells; however, it rarely infects the CNS. CMV plays a detrimental role in autoimmune neuroinflammation by enhancing the activation of disease-specific CD4+ T cells and aggravating autoimmunity-mediated inflammation and demyelination (4). Activated autoreactive immune cells infiltrate the brain and spinal cord, leading to chronic inflammation, demyelination, and ultimately, axonal loss (16). CMV may lead to CNS abnormalities including hearing and vision impairment, epilepsy, and disorders of motor and cognitive function (17).

To the best of our knowledge, this is the first report of a female with T-lymphoblastic lymphoma/ leukemia after allo-HSCT who had CMV infection after transplantation and subsequently developed AE. This patient underwent allo-HSCT with T-cell depletion *in vivo* using rabbit anti-human thymocyte immunoglobulin and immunosuppression, which may have contributed to the development of encephalitis. Several studies have reported cases of severe and recurrent anti-NMDA-type GluR and anti-GAD antibody-associated AE after HSCT due to abnormal reconstitution of the immune system by conditioning regimens (18, 19). However, severe CMV infection, another major risk factor, may disrupt tolerance and induce AE. Xu et al. reported a case of CMV infection-associated encephalitis causing confusion, fatigue, and impaired memory. The authors suggested that one possible reason for the critical course of CMV infection in their patient might be that they lacked a functional spleen, a condition previously associated with severe CMV infection (20).

Interestingly, in our case, CMV and antineuronal autoantibodies were not present in the CSF. We presumed that a prodromal period existed that preceded the characteristic onset of AE induced by CMV infection after allo-HSCT, which was presumed to contribute to the onset of encephalitis. Findings of our case are consistent with those of a large-scale analysis of herpesvirus DNA and antineuronal autoantibodies in the CSF of 113 patients with clinical signs of AE. The study showed that no autoantibodies were detected in 65 patients (57%), while 110 patients (97%) tested negative for the virus (21). Other viral infections may also be associated with encephalitis after transplantation. BK virus infection-induced encephalitis and end-stage renal disease was reported in a child with allo-HSCT. Authors speculated that in immunocompromised patients, viral reactivation may be associated with CNS involvement, leading to encephalitis (22).

In general practice, once severe invasive CMV disease is confirmed, immunosuppression should be avoided as much as possible to restore immune function and antiviral therapy should be initiated. In this case, the dose of cyclosporine was reduced and antiviral therapy with ganciclovir was initiated immediately after making the CMV diagnosis. Most patients have a good clinical response to antiviral therapy within 1–3 weeks of treatment initiation (15). However, this finding is inconsistent with our case, which required 1 month for CMV-DNA to slowly become undetectable.

This case report highlights AE involvement in a patient after allo-HSCT for invasive CMV, which was treated with a combination of high-dose pulsed methylprednisolone and IVIg therapies (23), resulting in a dramatic improvement of the patient’s neurological status. One year after admission, the patient exhibited nearly complete recovery, with mild deficits in attention and memory. A key observation that links active CMV infection after allo-HSCT and associated AE to the patient’s overall clinical presentation is that the use of combined treatment, including antivirals, immunomodulatory IVIg, and methylprednisolone, coincided with a rapid improvement in the patient’s clinical status (Figure 2). Early aggressive treatment (first-line immunotherapy) is associated with improved functional outcomes (24). However, assessing whether maintenance therapy with regular IVIg is needed is challenging. No reports exist on the efficacy of maintenance IVIg treatment for treating autoimmune neurological disorders. In addition to therapy for AE, checking for the development of comorbidities is important (25).

Pre-emptive monitoring and early treatment of CMV infections are critical for at-risk HSCT populations. The anti-CMV drug, letermovir, was recently approved for primary prophylaxis in HSCT recipients (26). However, this study provides a new strategy for universal primary prophylaxis in this population. Further interventional studies regarding CMV immune monitoring of the HSCT population are required (27). Despite the wide implementation of preventive strategies, studies have indicated a potential association between CMV replication (particularly early after transplantation) and adverse reactions after transplantation, even in the current era (28). The precise mechanisms underlying these adverse reactions remain unknown.

To conclude, the patient described herein developed clinical signs of AE after allo-HSCT, which may have been associated with immunosuppression after transplantation and subsequent CMV infection. Immunosuppression and CMV infection may be major risk factors for AE following allo-HSCT. Our main hypothesis is that allo-HSCT was the first insult to the patient’s immune status (immunosuppression after transplantation). Subsequently, the patient underwent a fulminant course of AE due to intractable CMV infection because immunological tolerance was disrupted, allowing autoimmunity to ensue. This report indicates that a combination of antiviral therapy; high-dose, pulsed methylprednisolone; and IVIg (provided as first-line therapy) may be an effective treatment option for CMV-associated AE after transplantation. We propose that the emerging concept of CMV-associated AE should be included in the differential diagnostic algorithm for patients presenting with signs of viral encephalitis because symptom overlap can occur. However, immune dysregulation in encephalitis and the relationship between CMV infection and AE after allo-HSCT require further study.

## Data availability statement

The original contributions presented in the study are included in the article/Supplementary material, further inquiries can be directed to the corresponding author.

## Ethics statement

The studies involving humans were approved by the ethics committee of Shanghai General Hospital. The studies were conducted in accordance with the local legislation and institutional requirements. The participants provided their written informed consent to participate in this study. Written informed consent was obtained from the individual(s) for the publication of any potentially identifiable images or data included in this article.

## Author contributions

MY: Writing – original draft, Writing – review & editing. YC: Writing – review & editing. LW: Writing – review & editing. LJ: Writing – review & editing. XS: Conceptualization, Writing – review & editing.
