# Supplementary material for: Case report: Autoimmune encephalomyelitis following cytomegalovirus infection after allogeneic hematopoietic stem cell transplantation
Source: Front Med (Lausanne). 2024 May 30;11:1373062. doi: 10.3389/fmed.2024.1373062 (PMC11169715; doi:10.3389/fmed.2024.1373062)
Supplement: Supplementary file 1 [file Data_Sheet_1.docx]

Supplementary Material

Case Report: Autoimmune Encephalomyelitis Following Cytomegalovirus Infection After Allogeneic Hematopoietic Stem Cell Transplantation

Running title: Post-CMV autoimmune encephalomyelitis post-allo HSCT

Min Yang, Yu Cai, Liping Wan, Linhua Ji, Xianmin Song

*** Correspondence:**Xianmin Song
[shongxm@sjtu.edu.cn](mailto:shongxm@sjtu.edu.cn)

**Supplementary Table 1**：The patient's previous chemotherapy regimen.

| Number | Chemotherapy regimen | Specific chemotherapy regimen |
| --- | --- | --- |
| 1 | VDPCP | Vindesine Sulfate 4mg d18,15,22 and Cyclophosphamide 1252mg d1, Idarubicin Hydrochloride 15mg d1-2, 10mg d3, Methylprednisolone Sodium Succinate 60mg d1-14, Pegasparagase 3750IU d8 |
| 2 | L-Hyper CVAD-A | Cyclophosphamide 465mg q12h*6 d1-3, Pegasparagase 3750u d2, Vindesine Sulfate 4mg d4. 11, Dexamethasone 40mg d1-4, 11-14, Epirubicin Hydrochloride 176mg d4 |
| 3 | Hyper CVAD B | Methotrexate 1.46g d1, Cytarabine 2.2g q12h d2-3 |
| 4 | IM2 | Ifosfamide 2g d1-5, Methotrexate 2g d1, Idarubicin, Hydrochloride 15mg d1, 10mg d2-3 |
| 5 | Hyper CVAD B | Methotrexate 1.5g d1, Cytarabine 3.5g q12h d2-3 |
| 6 | IM2 | Ifosfamide 1.5g d1-5, Methotrexate 1.5g d1, Idarubicin, Hydrochloride 10mg d1-3 |

**Supplementary Table 2**：Summary of the histocompatibility and immunogenetics between the patient and the donor.

| HLA | A | | B | | C | | DRB1 | | DQB1 | | DPB1 | |
| --- | --- | --- | --- | --- | --- | --- | --- | --- | --- | --- | --- | --- |
| Patient | 24:02 | 33:03 | 51:01 | 54:01 | 01:02 | 07:02 | 04:03 | 09:01 | 03:03 | 03:05 | 02:01 | 03:01 |
| Donor | 24:02 | 33:03 | 51:01 | 57:01 | 06:02 | 07:02 | 04:03 | 07:01 | 03:03 | 03:05 | 03:01 | 09:01 |

HLA, human leukocyte antigen.
